# Associations of systemic inflammatory markers with the risks of chronic heart failure: A case-control study

**DOI:** 10.1016/j.clinsp.2022.100056

**Published:** 2022-06-14

**Authors:** Zhaojun Liu, Yingjie Xv, Xiaozhu Liu, Xiaoli Zhou

**Affiliations:** a Department of Cardiology, The First Affiliated Hospital of Chongqing Medical University, Chongqing, China; b Department of Urology, The First Affiliated Hospital of Chongqing Medical University, Chongqing, China; c Department of Cardiology, The Second Affiliated Hospital of Chongqing Medical University, Chongqing, China

**Keywords:** Chronic heart failure, Inflammation, Systemic inflammatory indicators, Diagnosis, CHF, chronic heart failure, HF, heart failure, RAAS, renin-angiotensin aldosterone system, NLR, neutrophil-to-lymphocyte ratio, PLR, platelet-to-lymphocyte ratio, LMR, lymphocyte-to-monocyte ratio, MHR, monocyte-to-high-density-lipoprotein ratio, SII, systemic immune inflammation index, SIRI, system inflammation response index, LVEF, left ventricular ejection fraction, BNP, natriuretic peptides, BMI, body mass index, BUN, blood urea nitrogen, TC, total cholesterol, TG, triglycerides, HDL, high-density lipoprotein, LDL, low-density lipoprotein, hs-CRP, high sensitivity C reactive protein, CHD, coronary atherosclerotic heart disease, PSM, propensity score matching, ORs, odds ratios, CIs, confidence intervals, AUC, area under the curve, ROC, receiver operator characteristics curves, IL-1, interleukin 1, TNF-α, tumor necrosis factor-α

## Abstract

**Objective:**

As a greater proportion of patients survived their initial cardiac insult, Chronic Heart Failure (CHF) is becoming a major cause of worldwide morbidity and mortality. However, the mechanism underlying the inflammation in patients with CHF has not yet been elaborated. This study aims to explore the associations between inflammation and CHF patients, and the predictive performance of inflammatory indicators in identifying patients with CHF.

**Methods:**

A matched case-control study was conducted by recruiting 385 patients who were diagnosed with CHF from January 2018 to December 2019 in The First Affiliated Hospital of Chongqing Medical University. Each CHF patient was matched against one control subject without CHF on the criteria of age, sex, Body Mass Index (BMI), smoking status, and comorbidities. The clinical data and systemic inflammatory indicators were compared between the two groups, independent risk factors of CHF were identified by multivariate regression analysis, and the predictive values of systemic inflammatory indicators for CHF were analyzed by Receiver Operating Characteristic (ROC) curve analysis.

**Results:**

After processed in the univariate and multivariate regression analysis models, three systemic inflammatory indicators (hs-CRP [high sensitivity C Reactive Protein], LMR [lymphocyte-to-monocyte ratio], and Monocyte-to-High-density-lipoprotein Ratio [MHR]) were considered as independent predictors of CHF, among which the hs-CRP exhibited the best predictive performance (AUC = 0.752, 95%CI 0.717‒0.786, *p* < 0.001), followed by LMR (AUC = 0.711, 95% CI 0.675‒0.747, *p* < 0.001) and MHR (AUC = 0.673, 95% CI 0.635‒0.710, *p* < 0.001). The three-indicator combination showed an improved diagnostic performance (AUC = 0.757, 95% CI 0.724‒0.791, *p* < 0.001). In addition, the results of subgroup comparisons demonstrated that hs-CRP and MHR were associated with the severity of CHF (*p* < 0.001).

**Conclusions:**

The systemic inflammatory indicators such as hs-CRP, LMR, and MHR were independently correlated with the attack of CHF and might be the complementary markers of the diagnosis of CHF.

## Highlights

• Two novel inflammation-related markers, LMR and MHR are associated with Chronic Heart Failure (CHF).
• LMR and MHR were first proposed to be the predictors of a diagnosis of CHF in this study, which suggested that inflammation was associated with CHF, and anti-inflammation therapy might be a potential target for future therapeutic interventions.
• Compared with special inflammatory indicators such as TNF or IL-1, LMR and MHR are routinely measured in clinical practice and less time-consuming, which makes them suitable for popularization.

## Introduction

As the end stage of various cardiac diseases, Chronic Heart Failure (CHF), is a complex syndrome characterized by the inability of the heart to meet the metabolic demands of the body.^1^ According to the 2018 American Heart Association heart disease and stroke statistics update, the incidence of Heart Failure (HF) in the United States was 2.4% and would be increased distinctly.^2^ Even though most patients with CHF survived their initial cardiac insult, the heart failure inevitably develops to an acute episode, which arises after repeated and prolonged hospitalization.^3^ Given this, CHF is going to be a major cause of worldwide morbidity and mortality, and medical systems worldwide are being faced with a great challenge.^4^ Early detection and management of HF are of great importance to reduce admission rates and mortalities.

The developing mechanisms of HF include hemodynamic disorder, activation of the sympathetic nervous system and Renin-Angiotensin-Aldosterone System (RAAS), the cytokine hypothesis, and so on.^5^^,^^6^ Inflammation has been recognized as another possible mechanism of HF in recent years. Previous studies have demonstrated that the activation of classic neurohormonal systems and hemodynamic overload can trigger sustained myocardial inflammatory responses, resulting in the impairment of heart function.^7^ Researchers also have found that the magnitude in the elevation of proinflammatory cytokines in CHF is significantly less than what would be observed in cases of autoimmune diseases or acute infections, suggesting that low-grade chronic inflammation persists in CHF and may be an important contributor to the maintenance or clinical deterioration of patients with CHF.^8^^,^^9^ But recent clinical trials on anti-inflammatory therapy targeting for identified inflammatory indicators (such as TNF-α and IL-1) have not yielded satisfactory results, reflecting the insufficient understanding of the complex inflammatory networks within the heterogeneous syndrome of HF.^9^^,^^10^ Therefore, it is necessary to find novel inflammatory biomarkers to assist in identifying patients with HF who can benefit from anti-inflammatory therapy and reduce their prognostic risk.

Several systemic inflammatory indicators, including Lymphocyte-Monocyte Ratio (LMR), Platelet-Lymphocyte Ratio (PLR), Neutrophil-to-Lymphocyte Ratio (NLR), Monocyte-to-High-density-lipoprotein Ratio (MHR), Systemic Immune Inflammation Index (SII), and Systemic Inflammation Response Index (SIRI) have attracted much attention in recent years. These markers are simple to calculate according to routine blood indicators and have the advantage of being inexpensive and easy to detect, which thus have been used in many clinical studies for early assessment of the prognostic risk of various cardiovascular diseases.^11^, ^12^, ^13^ This study aims to explore the potential connections between inflammation and CHF, and the diagnostic value of systemic inflammatory indicators for CHF.

## Material and methods

### Study population

This retrospective study was approved by the Institutional Review Board of the First Affiliated Hospital of Chongqing Medical University and the requirement for patient informed consent was waived.

Data of patients with CHF were collected by using the electronic medical record system from January 2018 to December 2019 at the Department of Cardiology in the present study's institution. The diagnosis of CHF was determined by two or more experienced physicians based on the clinical history, specific clinical symptoms and signs, certain levels of B-type Natriuretic Peptides (BNP), and echocardiography.^14^ Patients with the following conditions were excluded: (1) With acute and chronic infection; (2) With end-stage liver disease or renal failure; (3) With hematological disorders or cancer; (4) With congenital heart disease; (5) With rheumatic immune system diseases; (6) Under glucocorticoids therapy (may affect coagulation); (7) absence of full-scale information. Finally, 755 of 1989 patients who met the inclusive and exclusive criteria were enrolled in the CHF group.

**Fig. 1:**
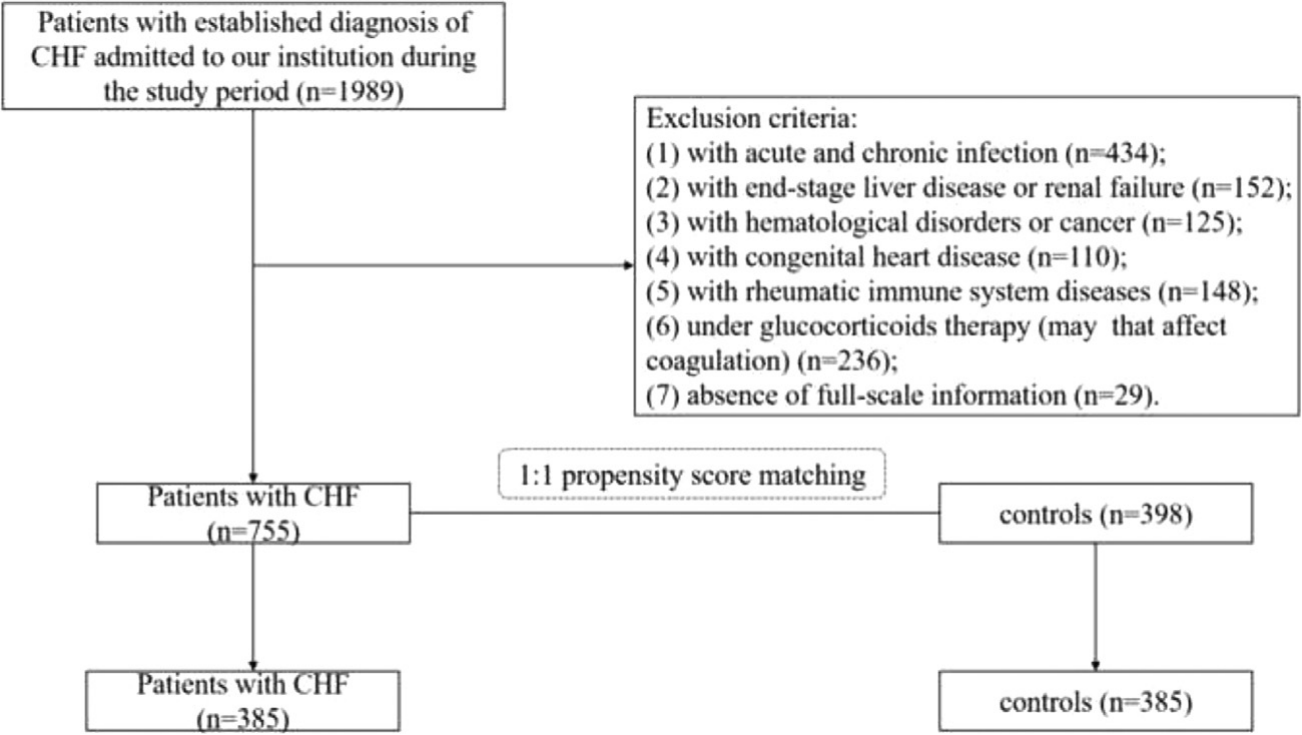
Flowchart of recruiting study cohorts. CHF, Chronic Heart Failure.

The control cohort was identified from an initial group of 398 continuous non-HF patients who were admitted to the present study's institute during the same period, of which, 385 subjects were confirmed to have normal findings on BNP and echocardiography and who were matched by age, gender, Body Mass Index (BMI), and comorbidities with the CHF group by 1:1 Propensity Score Matching (PSM). 385 pairs of patients were finally selected to set up the CHF group (*n* = 385) and the control group (*n* = 385). The flowchart of this study was shown in Fig. 1.

### Clinical and laboratory data collection

Demographic information including age, sex, BMI, smoking status, and preexisting comorbidities (diabetes mellitus, hypertension, and coronary atherosclerotic heart disease) was collected from the Electronic medical records in the institute. Blood samples were obtained on the first day of admission for the laboratory determination including BNP levels, biochemical parameters (Blood Urea Nitrogen [BUN], Creatine, Triglycerides [TG], Total Cholesterol [TC], High-Density Lipoprotein Cholesterol [HDL-C], Low-Density Lipoprotein Cholesterol [LDL-C], Apolipoprotein-A, Apolipoprotein-B, Lipoprotein(α) and high sensitivity C Reactive Protein [hs-CRP]), and blood routine test (platelet count, neutrophil count, lymphocyte count, and monocyte count). LVEF (Left Ventricular Ejection Fraction) assessment was based on 2D echocardiography using the quantitative 2D biplane volumetric Simpson method from 4- and 2-chamber views.

### Definitions

The authors summarized definitions of relative terms as follows:

(1) BMI was calculated as body weight (kg) divided by the square of height (m^2^);^15^
(2) Smoking status was defined as current tobacco use;
(3) Hypertension was defined as self-reported use of anti-hypertensive medication, having a history of hypertension, or Systolic Blood Pressure (SBP) ≥140 mmHg and/or Diastolic Blood Pressure (DBP) ≥ 90 mmHg;^16^
(4) Diabetes Mellitus (DM) was defined as self-reported physician diagnosis, fasting plasma glucose levels ≥7.0 mmoL/L, or use of oral hypoglycemic agents or insulin;^17^
(5) Coronary atherosclerotic Heart Disease (CHD) was defined as diameter stenosis of at least 50% in any of the major vessels in the coronary angiography image.^18^

### Calculations of inflammation-related indices

The systemic inflammation-related indices, including LMR, NLR, PLR, MHR, SII, and SIRI index were calculated using the following equations.

LMR=lymphocytecount/monocytecount

NLR=neutrophilcount/lymphocytecount

PLR=plateletcount/lymphocytecount

MHR=monocytecount/high−densitylipoproteincholesterol

SII=neutrophilcount×plateletcount/lymphocytecount

SIRI=neutrophilcount×monocytecount/lymphocytecount

### Propensity score matching

Matching ensures that the distributions of confounding variables are identical (or as close to identical as possible) so that the study group is comparable with the control group.^19^ This study used Propensity Score Matching (PSM) to reduce non-randomized selection bias and to reduce potential clinical confounders via a 1:1 matching protocol. Potential confounding covariates such as age, gender, BMI, smoking status, and comorbidities were included in this model. After adjustment of these confounders, Chi-Squared tests were performed to assess covariate balance between these two paired cohorts.

### Statistical analysis

Continuous variables were expressed as median (25th percentile, 75th percentile) for the skew distribution, and the Mann-Whitney *U* test was used to analyze the differences between groups. Categorical variables were described as numbers (percentages) and were assessed using the Chi-Square test or Fisher exact test.

Univariate analysis was performed to identify potential risk factors for CHF. Variables from the forward variable selection methods with a *p*-value of less than 0.1 were included in the multivariate logistic regression model to explore the independent risk factors. Odds Ratios (ORs) were reported with 95% Confidence Intervals (CIs) and *p*-values were calculated. The Area Under the Curve (AUC), specificity, and sensitivity of each indicator in the diagnosis of CHF patients were calculated by Receiver Operator Characteristics (ROC) curve analysis. Subsequently, two-indicator and three-indicator combinations were constructed to assess whether the predictive ability of CHF improved. Further comparisons were then performed to explore the significance of systemic inflammatory indicators for the severity of CHF, according to the BNP level (1st tertile: BN*p* < 287 pg/mL; 2nd tertile: 287 pg/mL ≤ BNP <1000 pg/mL; 3rd tertile ≥ 1000 pg/mL) and LVEF level (HFrEF: LVEF < 40%; HFmrEF: 40% ≤ LVEF < 50%, HFpEF: LVEF ≥ 50%). The model used in the subgroup analyses did not contain other covariates. All the statistical tests performed by the SPSS Statistics 26.0 were two-tailed, and a *p*-value of < 0.05 was considered as statistical significance.

## Results

### Baseline characteristics and systemic inflammatory indicators of the study cohorts

A total of 770 patients [male: female = 371 (48.2%): 399 (51.8%), the median age of 65 years (age range: 55‒75 years)] were included in the final cohorts and were divided into the CHF (385 patients) and control (385 controls) groups. 52.3% of the participants were diagnosed with hypertension, 23.1% suffered from coronary atherosclerotic heart disease, and 23.0% had diabetes mellitus. Additionally, 181 participants (23.5%) were current smokers. The distribution of the demographic characteristics of the included patients is summarized in Table 1.

**Table 1:** Demographic characteristics of patients with CHF and control subjects after PSM.

Variable	Post-matching			
	Total (*n* = 770)	Controls (*n* = 385)	Patients (*n* = 385)	*p-*value
**Demographic characteristics**				
Age (years)	65.00 (55.00, 75.00)	65.00 (55.00, 73.00)	66.00 (56.00, 76.00)	0.052
Gender (male), n (%)	371 (48.2)	181 (47.0)	190 (49.4)	0.516
BMI (kg/m^2^)	23.80 (21.60, 26.40)	23.90 (21.50, 26)	23.70 (21.60, 26.85)	0.412
**Comorbidities**				
Hypertension, n (%)	403 (52.3)	200 (52.0)	203 (53.0)	0.829
Diabetes mellitus, n (%)	177 (23.0)	84 (22.0)	93 (24.0)	0.441
Coronary atherosclerotic heart disease, n (%)	178 (23.1)	81 (21.0)	97 (25.0)	0.171
Smoking status, n (%)	181 (23.5)	88 (22.9)	93 (24.2)	0.671

Notes: Data were shown as s a number (%) or median (low quartile, upper quartile).

Abbreviations: BMI, body mass index; CHF, chronic heart failure; PSM, propensity score matching.

**Fig. 2:**
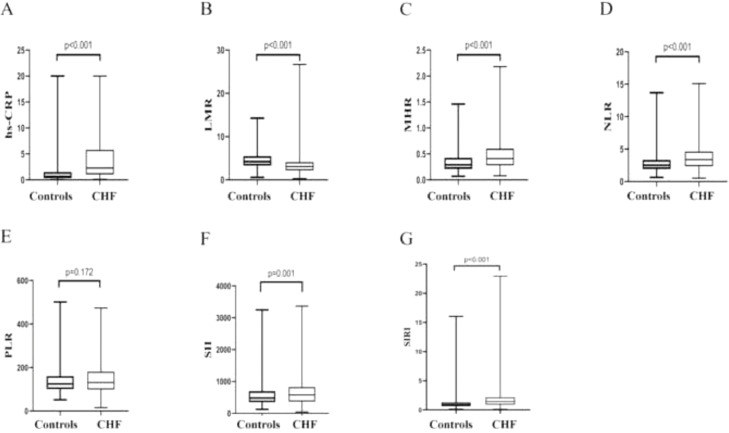
Comparisons of inflammatory indicators between CHF group and control group. Compared with control cohorts, the hs-CRP (*p* < 0.001), MHR (*p* < 0.001), NLR (*p* < 0.001), SII (*p* = 0.001), and SIRI (*p* < 0.001) levels of patients with CHF (A, B, C, D, F, G) were apparently increased, while the LMR levels of CHF patients were significantly decreased (B). There was no significant difference between two groups in terms of PLR levels (E). CHF, Chronic Heart Failure; hs-CRP, high sensitivity C Reactive Protein; LMR, Lymphocyte-to-Monocyte Ratio; MHR, Monocyte-to-High-density-lipoprotein Ratio; NLR, Neutrophil to Lymphocyte Ratio; PLR, Platelet-to-Lymphocyte Ratio; SII, Systemic Immune Inflammation Index; SIRI, System Inflammation Response Index.

Table 2 summarizes the comparisons of clinical and laboratory parameters between the two groups (after PSM). Compared with the control group, patients in the CHF group were significantly associated with more accounts of neutrophil (*p* < 0.001) and monocyte (*p* < 0.001), a fewer accounts of lymphocyte (*p* < 0.001), platelet (*p* < 0.001), meanwhile, higher level of BNP (*p* < 0.001), BUN (*p* < 0.001), creatinine (*p* < 0.001), and lower level of LVEF (*p* < 0.001), TC (*p* < 0.001), HDL-C (*p* < 0.001), LDL-C (*p* < 0.001) and apolipoprotein-A (*p* < 0.001). Systemic inflammatory indicators as hs-CRP (*p* < 0.001), LMR (*p* < 0.001), MHR (*p* < 0.001), NLR (*p* < 0.001), SII (*p* = 0.001), and SIRI (*p* < 0.001) were found contrasting between the two groups, except for PLR (Fig. 2). There was no significant difference between the CHF and non-HF groups in terms of TG, apolipoprotein-B, and lipoprotein (α).

**Table 2:** Baseline characteristics of participants with and without CHF after PSM.

Variable	Total (*n* = 770)	Controls (*n* = 385)	Patients (*n* = 385)	*p-*value
BNP (pg/mL)	87.75 (38.10, 596.00)	38.30 (26.50, 49.05)	596.00 (229.00, 1163.00)	< 0.001
LVEF (%)	59.00 (44.00, 64.00)	63.00 (59.00, 66.00)	45.00 (35.00, 64.00)	< 0.001
Neutrophil count (10^9^/L)	4.14 (3.26, 5.10)	3.91 (3.12, 4.72)	4.39 (3.47, 5.31)	< 0.001
Lymphocyte count (10^9^/L)	1.45 (1.07, 1.83)	1.55 (1.19, 1.90)	1.33 (0.99, 1.71)	< 0.001
Monocyte count (10^9^/L)	0.39 (0.30, 0.50)	0.35 (0.28, 0.45)	0.43 (0.34, 0.56)	< 0.001
Platelet count (10^9^/L)	182.50 (148.00, 223.25)	195.00 (158.00, 232.00)	173.00 (136.00, 214.00)	< 0.001
BUN (mg/dL)	6.00 (5.00, 7.70)	5.50 (4.70, 6.45)	7.00 (5.50, 9.10)	< 0.001
Creatinine (μmoL/L)	74.00 (62.75, 93.00)	71.00 (61.00, 84.00)	79.00 (64.50,101.00)	< 0.001
TC (mmoL/L)	3.80 (3.23, 4.30)	3.94 (3.42, 4.41)	3.58 (3.01, 4.19)	< 0.001
TG (mmoL/L)	1.10 (0.82, 1.46)	1.13 (0.85, 1.52)	1.06 (0.80, 1.41)	0.09
HDL-C (mmoL/L)	1.15 (0.93, 1.42)	1.23 (1.02, 1.48)	1.05 (0.85, 1.37)	< 0.001
LDL-C (mmoL/L)	2.26 (1.76, 2.72)	2.38 (1.85, 2.80)	2.09 (1.69, 2.61)	< 0.001
Apolipoprotein-A (g/L)	1.29 (1.10, 1.49)	1.40 (1.23, 1.54)	1.17 (0.97, 1.38)	< 0.001
Apolipoprotein-B (g/L)	0.77 (0.63, 0.91)	0.79 (0.63, 0.90)	0.76 (0.63, 0.93)	0.82
Lipoprotein (α) (mg/L)	80.50 (38.00, 186.25)	76.00 (35.50, 171.00)	86.00 (39.00, 209.00)	0.12

Notes: Data were shown as median (low quartile, upper quartile).

Abbreviations: CHF, chronic heart failure; PSM, propensity score matching; BNP, brain natriuretic peptide; LEVF, left ventricular ejection fraction; BUN, blood urea nitrogen; TC, total cholesterol; TG, triglyceride; HDL-C, high-density lipoprotein cholesterol; LDL-C, low-density lipoprotein cholesterol.

### Univariate and multivariate logistic regression analysis

Logistic regression analysis was conducted to evaluate the associations between inflammatory indicators and CHF. Variables with statistical significance in the univariate logistic regression analysis were then processed in multivariate logistic regression analysis to assess their predictive significance for CHF. As listed in Table 3, hs-CRP (OR = 1.125, 95% CI 1.074‒1.179, *p* < 0.001), LMR (OR = 0.802, 95% CI 0.724‒0.887, *p* < 0.001), MHR (OR = 5.288, 95% CI 2.080‒13.447, *p* < 0.001), platelet (OR = 0.994, 95% CI 0.992‒0.997, *p* < 0.001) and BUN (OR = 1.461, 95% CI 1.329‒1.607, *p* < 0.001) were proved to be the independent risk factors of CHF.

**Table 3:** Multivariate analyses of related factors for CHF in the study cohort.

Variables	Multivariate analysis	
	OR (95% CI)	*p*-value
Hs-CRP	1.125 (1.074‒1.179)	< 0.001
LMR	0.802 (0.724‒0.887)	< 0.001
MHR	5.288 (2.080‒13.447)	< 0.001
Platelet count	0.994 (0.992‒0.997)	< 0.001
BUN	1.461 (1.329‒1.607)	< 0.001

Note: Data are given for univariate and multivariate regression model.

Abbreviations: CHF, chronic heart failure; CI, confidence intervals; hs-CRP, high sensitivity C reactive protein; LMR, lymphocyte-to-monocyte ratio; MHR, monocyte-to-high-density-lipoprotein ratio; BUN, blood urea nitrogen; OR, odds ratio.

### Predictive values of systemic inflammatory indicators for patients with CHF

**Fig. 3:**
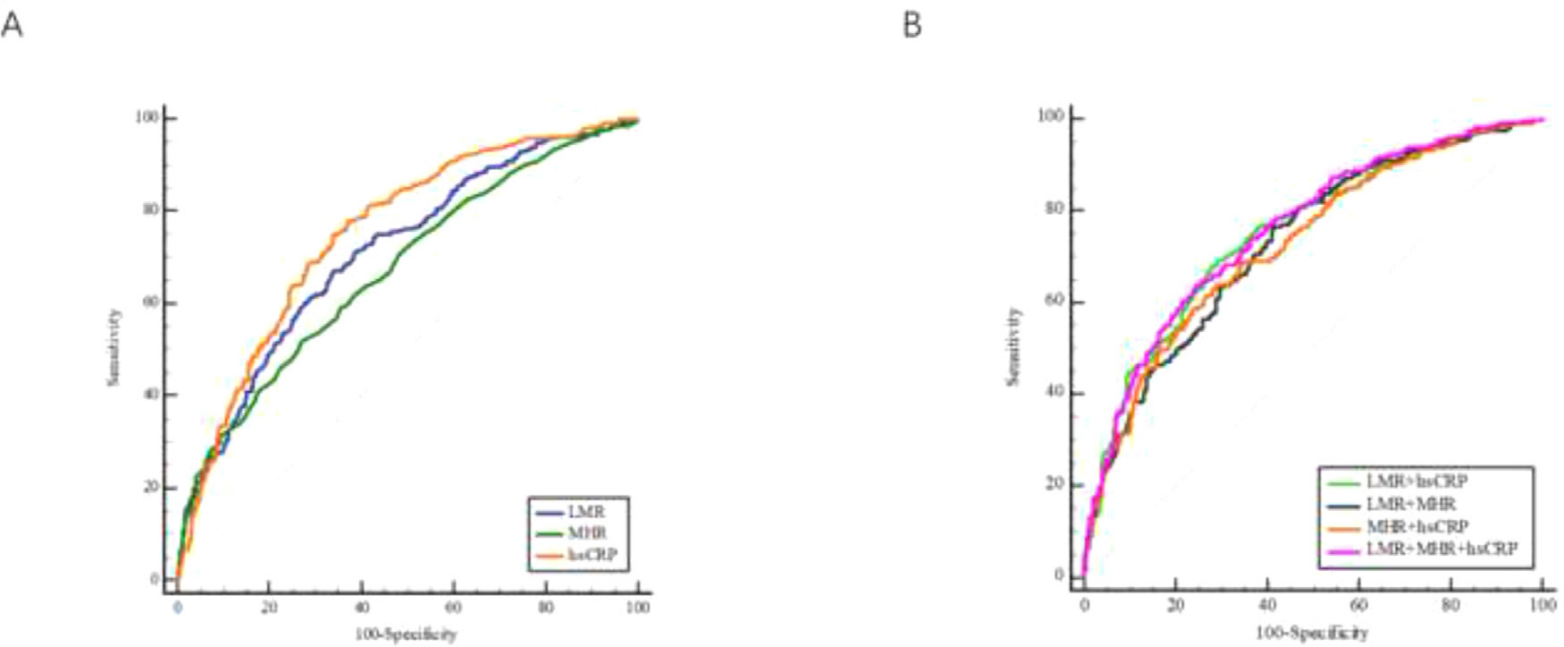
The predictive performance of LMR, MHR, and four combinations in diagnosis of patients with CHF. ROC curves of LMR, MHR, and hs-CRP (A). ROC curves of the four combinations (B). CHF, Chronic Heart Failure; hs-CRP, high sensitivity C Reactive Protein; LMR, Lymphocyte-to-Monocyte Ratio; MHR, Monocyte-to-High-density-lipoprotein Ratio; ROC, Receiver Operator Characteristics.

ROC curve analysis was employed to evaluate the predictive abilities of systemic inflammatory indicators. Of the three systemic inflammatory indicators, hs-CRP (AUC = 0.752, 95% CI 0.717‒0.786, *p* < 0.001) exhibited the best diagnostic performance with a sensitivity of 0.779 and a specificity of 0.629, while LMR and MHR achieved AUC values of 0.711 (95% CI 0.675‒0.747, *p* < 0.001) and 0.673 (95% CI 0.635‒0.710, *p* < 0.001), respectively (Fig. 3A). The predictive performances of each indicator are listed in Table 4.

**Table 4:** Predictive performances of hs-CRP, LMR, MHR and four combinations for CHF.

Variable	AUC	95% CI	p*-*value	Cutoff value	Sensitivity	Specificity
Hs-CRP	0.752	0.717‒0.786	< 0.001	0.940	0.779	0.629
LMR	0.711	0.675‒0.747	< 0.001	3.710	0.670	0.660
MHR	0.673	0.635‒0.710	< 0.001	0.400	0.517	0.733
LMR + MHR	0.730	0.694‒0.765	< 0.001	/	0.761	0.590
LMR + hs-CRP	0.751	0.717‒0.785	< 0.001	/	0.678	0.722
MHR + hs-CRP	0.727	0.692‒0.762	< 0.001	/	0.639	0.709
LMR + MHR+ hs-CRP	0.757	0.724‒0.791	< 0.001	/	0.647	0.743

Abbreviations: AUC, area under the curve; CHF, chronic heart failure; CI, confidence intervals; hs-CRP, high sensitivity C reactive protein; LMR, lymphocyte-to-monocyte ratio; MHR, monocyte-to-high-density-lipoprotein ratio.

To explore a better predictive performance, combinations of two indicators and three indicators were established. The AUC, *p*-value, 95% CI, sensitivity, and specificity were calculated to evaluate their performances (Table 4 and Fig. 3B). Among the two-indicator combinations, LMR combined with hs-CRP presented the best predictive performance, with an AUC of 0.751 (95% CI 0.717‒0.785, *p* < 0.001), a sensitivity of 0.678, and a specificity of 0.722. The three-indicator combination (hs-CRP, LMR and MHR) showed improved performance, obtaining a greater AUC (0.757, 95% CI 0.724‒0.791, *p* < 0.001) and specificity (specificity = 0.743).

### Subgroup analysis

**Fig. 4:**
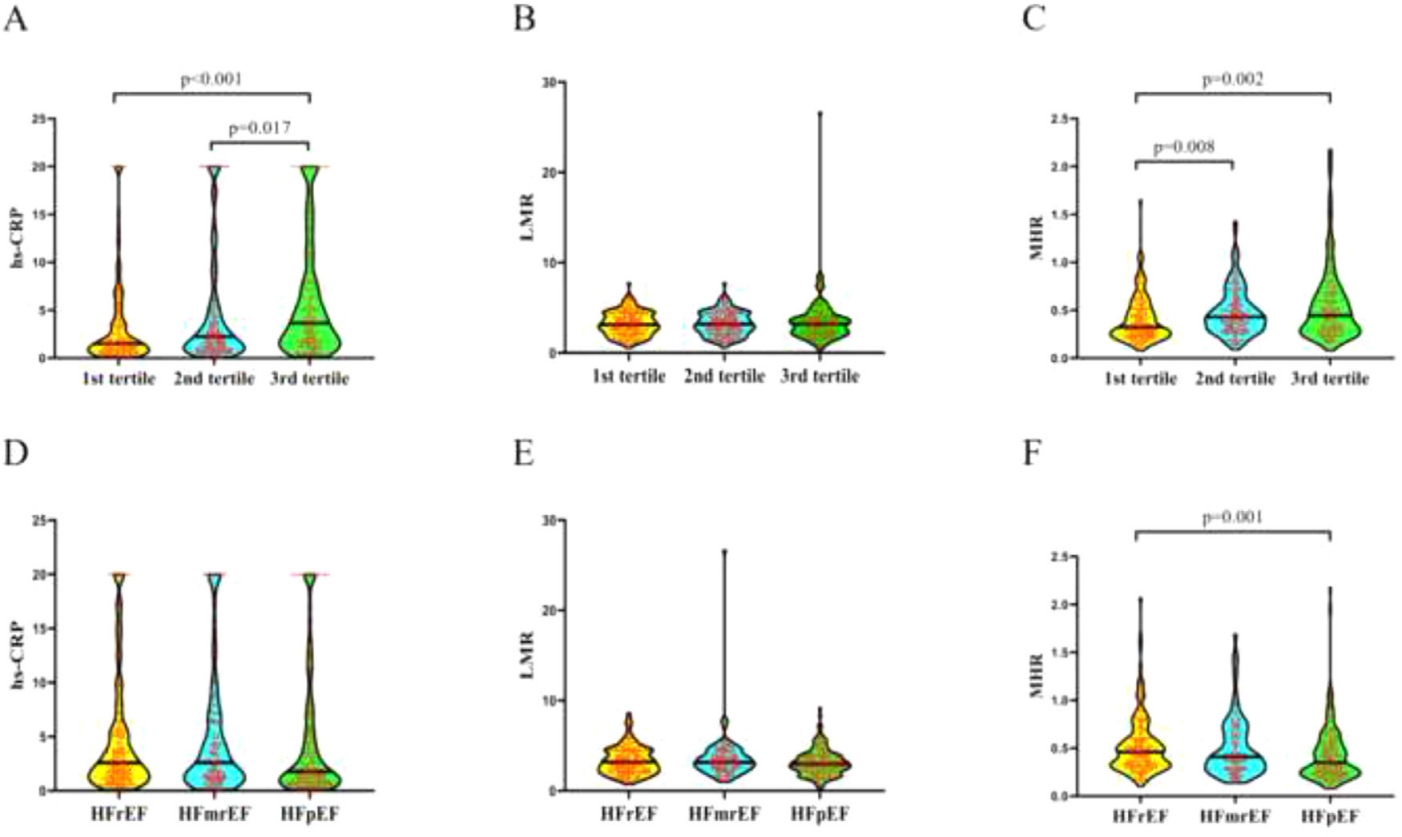
Comparisons of hs-CRP, LMR and MHR among different groups according to BNP levels (A‒C) and LVEF levels (D‒E). The hs-CRP values of patients in 3^rd^ tertile were significantly higher than 1^st^ tertile group (*p* < 0.001) and 2^nd^ group (*p* = 0.017) (A); the MHR values of patients in 1^st^ tertile group were significantly lower than 2^nd^ tertile group (*p* = 0.008) and 3^rd^ tertile group (*p* = 0.002) (C); the values of MHR in HFrEF group were significantly higher compared to HFpEF group (*p* < 0.001) (F). No significant difference was found between either LMR and LVEF, nor LMR and BNP. (B, D, E). BNP, Brain Natriuretic Peptide; HFrEF, Heart Failure with reduced Ejection Fraction; HFmrEF, Heart Failure with mid-range eEjection Fraction; HFpEF, Heart Failure with preserved Ejection Fraction; hs-CRP, high sensitivity C Reactive Protein; LVEF, Left Ventricular Ejection Fraction; LMR, Lymphocyte-to-Monocyte Ratio; MHR, Monocyte-to-High-density-lipoprotein Ratio.

The patients with CHF were divided into subgroups according to BNP and LVEF levels. To explore the underlying associations of the systemic inflammatory indicators with the development of CHF, the Mann-Whitney *U* test was performed to determine whether there were differences in terms of the hs-CRP, LMR, and MHR among different subgroups (Tables 5 and 6). As shown in Fig. 4, compared with the 1^st^ group and 2^nd^ tertile group, increased hs-CRP values were found in 3^rd^ tertile (*p* < 0.001 and *p* = 0.017, separately), while patients in the 1^st^ tertile group exhibited decreased MHR levels than that of patients in 2^nd^ tertile group (*p* = 0.008) and 3^rd^ tertile group (*p* = 0.002). Besides, compared with HFrEF group, patients in HFpEF group had lower MHR values (*p* < 0.001). Except for that, there was no statistical difference in LMR among different BNP and LVEF subgroups

**Table 5:** Comparisons of different level of LVEF with inflammation-related indicators in patients with CHF.

Variable	Total (*n* = 385)	HFrEF (*n* = 141)	HFmrEF (*n* = 90)	HFpEF (*n* = 154)	*p*-value
hs-CRP	2.280 (1.050, 5.805)	2.590 (1.240, 5.565)	2.610 (1.138, 6.413)	1.780 (0.820, 5.755)	0.153
LMR	3.070 (2.195, 4.120)	3.190 (2.135, 4.440)	3.170 (2.460, 4.323)	2.990 (2.145, 3.873)	0.242
MHR	0.410 (0.280, 0.600)	0.460 (0.320, 0.605)	0.410 (0.280, 0.663)	0.350 (0.238, 0.553)	0.001

Note: HFrEF: LVEF < 40%; HFmrEF: 40% ≤ LVEF 50%, HFpEF: LVEF ≥ 50%.

Abbreviations: CHF, chronic heart failure; hs-CRP, high sensitivity C reactive protein; LVEF, left ventricular ejection faction; LMR, lymphocyte-to-monocyte ratio; MHR, monocyte-to-high-density-lipoprotein ratio.

**Table 6:** Comparisons of different level of BNP with inflammation-related indicators in patients with CHF.

Variable	Total (*n* = 385)	1st tertile (*n* = 128)	2nd tertile (*n* = 127)	3rd tertile (*n* = 130)	*p*-value
hs-CRP	2.280 (1.050, 5.805)	1.530 (0.735, 3.850)	2.240 (0.940, 5.270)	3.665 (1.648, 7.910)	< 0.001
LMR	3.070 (2.195, 4.120)	2.965 (2.205, 4.030)	3.190 (2.170, 4.360)	3.200 (2.195, 3.990)	0.803
MHR	0.410 (0.280, 0.600)	0.325 (0.240, 0.520)	0.430 (0.300, 0.610)	0.445 (0.290, 0.680)	0.001

Note: 1^st^ tertile: BNP < 287 pg/mL; 2^nd^ tertile: 287 pg/Ml ≤ BNP ≤ 1000 pg/mL; 3^rd^ tertile ≥ 1000 pg/mL.

Abbreviations: BNP, Brain Natriuretic Peptide; CHF, Cronic Heart Failure; hs-CRP, high sensitivity C Reactive Protein; LMR, Lymphocyte-to-Monocyte Ratio; MHR, Monocyte-to-High-density-lipoprotein Ratio.

## Discussion

The present study discovered that the systemic inflammatory indexes including hs-CRP (*p* < 0.001), LMR (*p* < 0.001), and MHR (*p* < 0.001) were independently associated with the development of CHF, and presented satisfying diagnostic values, with AUC values of 0.752, 0.711, and 0.673, respectively. The three-indicator model (hs-CRP, LMR, and MHR) revealed the best predictive performance (AUC = 0.757, 95% CI 0.724‒0.791, *p* < 0.001). In addition, elevated hs-CRP (*p* < 0.001) and MHR (*p* = 0.001) were found in patients with a more serious CHF. These findings demonstrate that the inflammatory mechanism plays an important role in CHF, and inflammatory indicators (such as hs-CRP, LMR, and MHR) may be the complementary biomarkers in the diagnosis of CHF.

The correlations between inflammation and HF have been widely discussed in recent years.^20^, ^21^, ^22^ Several studies have reported that the myocardial injury of patients with HF would activate the innate and adaptive immune systems, which is the trigger of the systemic inflammatory state.^7^ Subsequently, proinflammatory cytokines and chemokines increased, along with the neutrophils and monocytes infiltrated into the injured myocardium, which provided a short-term adaptation to stress in the heart, named physiologic inflammation.^23^ If myocardial damage persisted, prolonged inflammation would lead to left ventricular dysfunction and remodeling.^24^ In addition, in the acute heart injury models, researchers have clearly found that inflammatory cells play an important role in the pathogenesis of Acute Heart Failure (AHF).^25^ Previous studies have found that several systemic inflammatory indicators are independent risk factors in the prognosis of patients with AHF.^26^^,^^27^ The systemic inflammatory mechanism in patients with AHF is relatively clear. But the role of systemic inflammatory indicators (such as LMR and MHR) in CHF have not yet been elaborated. This study aimed to explore the role of systemic inflammatory indicators plays in CHF, for the better development of potential therapeutic targets and formulation of risk reduction strategies that match individual risk levels.

Increased serum CRP level was found to be a prognostic biomarker of patients with CHF.^28^^,^^29^ The present results were in accordance with the former studies, and extended it as the hs-CRP not only is an independent risk factor of CHF (*p* < 0.001), but also yielded a preferable diagnostic performance (AUC = 0.752). In contrast, although the previous study demonstrated that NLR and PLR have diagnostic values of HF (AUC value of 0.868 and 0.689, respectively), there was no consistent result obtained in the present study.^30^ A possible explanation was that the sample size in the previous study was insufficient to investigate the relationships with the risk for HF owing to its relatively low incidence. In addition, another report has revealed the associations between SII and SIRI and cardiovascular diseases and all-cause mortality, whereas ours did not come to the same conclusion.^13^

As for LMR, a simple and low-cost inflammatory marker, which is determined by the counts of lymphocytes and monocytes, is known to be a prognostic biomarker of patients with HF, malignant hematologic disorders, and malignant tumors.^31^^,^^32^ In the present study, LMR was proved to be an independent risk factor of CHF (*p* < 0.001), with superior diagnostic performance (AUC of 0.711, 95% CI 0.570‒0.6400). Differed from the LMR, MHR was calculated by monocytes and high-density lipoprotein. Even no previous evidence has indicated the associations between MHR and HF, as its’ components, monocytes and HDL-C have been broadly discussed in HF.^33^^,^^34^ Considered previous studies have proved that MHR was an independent prognostic marker for patients with malignant tumors and myocardial infarction, we wondered whether MHR was a potential diagnostic indicator for CHF.^35^^,^^36^ As a result, MHR proved to be a novel diagnostic indicator with an AUC value of 0.673 (95% CI 0.638‒0.706, *p* < 0.001), which is the first time. In view of these, the inflammatory indicators (hs-CRP, LMR and MHR) are associated with CHF, and the results of anti-inflammatory therapy targeting these markers might be promising.

To further explore the diagnostic values of inflammatory indicators for CHF, different combinations consisting of hs-CRP, LMR and MHR were constructed. Compared with single indicators or other two-indicator combinations, the three-indicator combination (hs-CRP, LMR, and MHR) possessed an improved diagnostic ability in predicting CHF (AUC = 0.757).

Subgroup comparisons according to BNP and LVEF levels were performed to explore the connection between inflammation and the severity of CHF. As a result, the elevated hs-CRP level was associated with increased BNP (*p* < 0.001), while was not correlated with LVEF, which may result from the unequal number of LVEF subgroups. Furthermore, patients in the HFrEF group presented higher MHR values than the HFpEF group (*p* = 0.001), and lower MHR values in the 1^st^ tertile group than 2^nd^ tertile group (*p* = 0.008) and 3^rd^ tertile group (*p* = 0.002), which indicated that the level of MHR was associated with the severity of CHF. These findings might provide clinical proof for exploring the inflammatory mechanism of CHF.

In summary, the present study differed from others in the following terms: (1) The authors replicated prior findings which suggested that inflammation was associated with CHF, and firstly proposed LMR and MHR as the complementary diagnostic markers in patients with CHF; (2) Compared with special inflammatory indicators such as TNF or IL-1, the systemic inflammatory markers in the present study are routinely measured in clinical practice, which makes them suitable for popularization and application; (3) To reduce selection bias, the PSM was employed to balance baseline characteristics between CHF and control groups, increasing the reliability level of evidence in the present study.

This study had several limitations, including the retrospective design, small sample size, and the lack the pathophysiological data to better clarify the mechanism of inflammation in CHF. Moreover, the authors have excluded patients with end-stage liver disease or renal failure, but the present indicators, the non-specific indicators reflecting systemic inflammation, are still affected by various diseases, which need further studies to address these issues.

## Conclusion

In conclusion, the present study indicated the association of systemic inflammatory indicators with CHF, and firstly proposed LMR and MHR as the independent predictive factors for patients with CHF, which might enrich the research field of predictors of CHF, introduce available indicators for risk management of HF and provide potential therapeutic targets for CHF.

## CRediT authorship contribution statement

**Zhaojun Liu:** Visualization, Investigation, Formal analysis, Writing – original draft, Writing – review & editing. **Yingjie Xv:** Project administration. **Xiaozhu Liu:** Project administration. **Xiaoli Zhou:** Visualization, Investigation.

## CRediT authorship contribution statement

**Zhaojun Liu:** Visualization, Investigation, Formal analysis, Writing – original draft, Writing – review & editing. **Yingjie Xv:** Project administration. **Xiaozhu Liu:** Project administration. **Xiaoli Zhou:** Visualization, Investigation.

## Declaration of Competing Interest

The authors declare that they have no known competing financial interests or personal relationships that could have appeared to influence the work reported in this paper.
